# Inflammation and Pancreatic Cancer: Focus on Metabolism, Cytokines, and Immunity

**DOI:** 10.3390/ijms20030676

**Published:** 2019-02-05

**Authors:** Andrea Padoan, Mario Plebani, Daniela Basso

**Affiliations:** Department of Medicine – DIMED, University of Padova, Via Giustiniani 2, 35128 Padova, Italy; E-Mails: andrea.padoan@unipd.it (A.P.); mario.plebani@unipd.it (M.P.)

**Keywords:** anti-TNFα, cytokines, inflammation, miRNA, myeloid derived suppressor cells, pancreatic cancer, S100A8, S100A9, TNFα, T_reg_ lymphocytes, tumor associated macrophages

## Abstract

Systemic and local chronic inflammation might enhance the risk of pancreatic ductal adenocarcinoma (PDAC), and PDAC-associated inflammatory infiltrate in the tumor microenvironment concurs in enhancing tumor growth and metastasis. Inflammation is closely correlated with immunity, the same immune cell populations contributing to both inflammation and immune response. In the PDAC microenvironment, the inflammatory cell infiltrate is unbalanced towards an immunosuppressive phenotype, with a prevalence of myeloid derived suppressor cells (MDSC), M2 polarized macrophages, and T_reg_, over M1 macrophages, dendritic cells, and effector CD4^+^ and CD8^+^ T lymphocytes. The dynamic and continuously evolving cross-talk between inflammatory and cancer cells might be direct and contact-dependent, but it is mainly mediated by soluble and exosomes-carried cytokines. Among these, tumor necrosis factor alpha (TNFα) plays a relevant role in enhancing cancer risk, cancer growth, and cancer-associated cachexia. In this review, we describe the inflammatory cell types, the cytokines, and the mechanisms underlying PDAC risk, growth, and progression, with particular attention on TNFα, also in the light of the potential risks or benefits associated with anti-TNFα treatments.

## 1. Introduction

Pancreatic ductal adenocarcinoma (PDAC), one of the deadliest cancers worldwide, has a five-year survival rate of less than 6%, the lowest percentage for cancers in the period 2007–2013 [1]. The incidence of PDAC estimated for the year 2018 is 2.5%, with over 18 million new cases being diagnosed worldwide, while mortality is estimated as being 4.5% of the 9.6 million cancer-related deaths [2]. This low survival rate is mainly attributable to late diagnosis, because PDAC is almost entirely asymptomatic until it becomes advanced. This limits the percentage of patients benefitting from surgical resection combined with adjuvant multi-agent chemotherapy, the current standard of care [3].

Unlike other types of cancer, few risk factors have been attributed to PDAC. Although geographical variations might exist between populations [2], cigarette smoking, diabetes mellitus, chronic pancreatitis, and obesity are the most relevant risk factors for this tumor [4,5,6,7,8], while some nutrients (i.e., folates) or drugs (i.e., aspirin and metformin) have a slight protective effect [5,9,10,11,12,13]. The incidence of PDAC also differs between sexes, currently being higher in women than in men [1], and is linked to PDAC family history and heavy alcohol consumption [14].

The potential role of inflammation in the development and growth of cancer was initially described in 1863 by Virchow [15], who observed that inflammatory cells infiltrate tumors. In recent years, the advancements made in cancer biology have confirmed that inflammation plays an important role in the development of PDAC and its progression. The persistence of inflammation, a process initially triggered to protect the organism against loss of tissue homeostasis, is involved in several steps of the carcinogenetic process [16]. Organ confined chronic inflammatory diseases are known to enhance the risk of colorectal cancer in patients with inflammatory bowel diseases, oesophageal cancer in patients with Barrett’s oesophagus, gastric cancer in patients with *H. pylori* infection [17,18,19], and pancreatic cancer in patients with chronic pancreatitis [6]. Systemic diseases characterized by low-grade chronic inflammation, such as metaflammation in patients with the metabolic syndrome, and diabetes mellitus, also enhance the risk of cancer, particularly pancreatic cancer [20]. Conversely, inflammation is chronically and abnormally present in cancer—the “wound that never heals”. In this respect, immune cell populations have been linked to several processes in the interactions between tumor stroma, cancer cells, and local inflammation occurring within the tumor microenvironment.

The present review highlights the potential role of inflammation in PDAC development and progression, focusing first on the interactions known to exist among immune cells, tumor-surrounding stroma, and tumor cells (local inflammation), and second, on the role of inflammatory cytokines. Inflammatory conditions, such as diabetes and obesity, are also investigated, with recently reported findings supporting the effects of systemic inflammation on PDAC being reivewed.

## 2. Role of Inflammation, Diabetes, and Obesity in Enhancing PDAC Risk

It is well known that longstanding diabetes mellitus and obesity are predisposing medical conditions for PDAC [5,7,8,21,22,23,24,25,26,27]. On the other hand, PDAC itself is known to cause diabetes mellitus by reducing insulin release, enhancing insulin resistance, and leading to new onset diabetes mellitus, which is diagnosed in more than 60% of patients with this tumor type [28,29].

Diabetes mellitus and obesity, which frequently coexist in the metabolic syndrome, are risk factors not only for PDAC, but also for other cancer types, as well as cardiovascular diseases. The chronic subclinical inflammation associated with the state of hyperadiposity or with diabetes mellitus, the so-called “metaflammation”, supports the concept that there is a link between chronic inflammation and cancer [20]. The majority of obese patients present a reduced release of the anti-inflammatory adipokine adiponectin and an increased release of the pro-inflammatory adipokine leptin. Moreover, a shift from the M2 anti-inflammatory to the M1 pro-inflammatory macrophages that infiltrate the adipose tissue, triggers the release of pro-inflammatory cytokines, mainly tumor necrosis factor alpha (TNFα) and interleukin (IL)-6 [30]. The metabolic syndrome might also enhance cancer risk through the increased release of the vascular endothelial growth factor (VEGF), a pro-angiogenetic cytokine, but also through the pro-proliferative effects of insulin, the levels of which are, in most cases, increased consequent to peripheral insulin resistance. As recently demonstrated by Wu et al. [31], glucose itself has a potential oncogenic effect, as it destabilizes the tumor suppressive function of ten-eleven translocation protein 2 (TET2) involved in DNA methylation.

Various complex mechanisms thus concur in favoring cancer development in an environment of chronic inflammation, but a primary role is played by inflammatory cells. Inflammation is closely correlated with immunity, the same immune cell populations contributing to both inflammation and immune response. As a consequence, a triad of phenomena are entwined in cancer, with inflammatory cells at the cross road, namely: (1) inflammatory cells govern inflammation, which might enhance cancer risk, and the same inflammatory cells govern the immune reaction against emerging cancer cells; (2) during cancer evolution, inflammatory cells might orchestrate the escape of tumor cells from immune control by modifying their antigenic fingerprint and favoring a shift towards a more immunosuppressive phenotype; and (3) inflammatory cells might affect, and be affected by, tumor cell metabolism, mainly glucose metabolism, and through this metabolic pathway, they might impact on tumor growth and escape from immune control [32,33,34]. Traditionally, the relation between cancer and the immune system has been summarized as the “3E hypothesis”, which is as follows: (1) elimination by the immune system cells of emerging cancer cells, (2) equilibrium between cancer cells and immune cells characterized by controlled tumor growth, and (3) escape of cancer cells from the immunosurveillance characterized by uncontrolled tumor growth [35]. The key player molecules involved in this dynamic and continuously evolving inflammatory and immunomodulatory scenario are cytokines produced by both tumor and inflammatory cells, including the pro-inflammatory IL-1β, IL-6, and TNFα; the anti-inflammatory IL-10; and the dual-face cytokine transforming growth factor beta (TGFβ), which exerts opposite and context-dependent effects. Cytokines are also implicated in linking inflammation and metabolism, and, in this context, it has been demonstrated that there is a highly conserved relationship between the pleiotropic immune mediator TNFα; the pathogen sensing toll like receptors (TLRs); and the metabolic hormone, insulin [20]. TNFα, produced by macrophages, may act as a metabolic hormone blocking insulin signaling or production, as TLRs do, but also as a regulator of the immune response, therefore being a representative molecule linking inflammation, metabolism, immune response, and cancer.

Patients with the metabolic syndrome might be at a higher risk of cancer, PDAC in particular, not only because of underlying metaflammation activating oncogenic pathways in epithelial cells while reducing immune cell response to cancer, but also because of the effects of other activated pathways linked to nutrition and gut-microbiota [16,36]. Overeating and an unhealthy diet may incur an excessive intake of nutrients and potential carcinogens. The role of diet in increasing PDAC risk has been evaluated in various epidemiological studies, all of which indicate the protective role of a diet rich in fruit, vegetables, whole grains, and lean meat, an increased risk being observed when the diet is rich in fatty meat, dairy fat, and sugar [10]. Nutrition quantity and quality might also impact on the intestinal microbiota, favoring dysbiosis with a reduced intestinal barrier function that collectively favors endotoxinemia, and an over-representation of the bacterial species producing pro-carcinogenic metabolites [16]. Overeating has a strong immunomodulatory effect since it leads to a vicious circle by generating a subclinical inflammatory status with increased levels of pro-inflammatory cytokines, associated with excessive glucose and lipid concentrations and increased trophic hormones. Diet might also impact on immune cells through the gut microbiota. Microorganisms produce numerous metabolites that affect the host metabolism and immunity, an example being short-chain fatty acids (SCFAs), whose profile depends on the type of bacteria present and dietary chemical composition (glucans vs. fructans vs. polyphenols). Microbial structural components and products may act on local epithelial cells, and at distant sites. Fatty acids, found in excess in patients with the metabolic syndrome, might impact on inflammatory cells, favoring the shift towards an immunosuppressive phenotype that allows for tumor cells to escape from immune control [30]. For example, butyrate, an SCFA, may be an energy source for colonocytes contributing to their renewal, but it favors the increased expression of the Foxp3 boosting T_reg_ function [37]. Moreover, the gut microbiome is a source of lipopolysaccharide (LPS), a major component of the cell wall of Gram-negative bacteria, which is able to trigger inflammation and insulin resistance by binding the TLR4 of immune cells and activating the NF-κB signaling pathway [38]. In patients with the metabolic syndrome, increased blood levels of LPS and of the most important LPS ligand, the LPS binding protein (LBP), have been described and found to be correlated with insulin resistance, further supporting the detrimental role of chronic inflammation in glucose metabolism [39,40,41]. Glucose metabolism is also crucial for established tumors, which are characterized by a pronounced glycolytic signature, the so-called Warburg effect [42]. Within the restricted tumor-microenvironment ecosystem, tumor cells might directly shape immune cells towards an immunosuppressive phenotype through metabolic competition and the direct or exosomes-mediated transfer of metabolites, enzymes, and nucleic acids [32,43].

## 3. The Role of Local Inflammation in PDAC Growth, Development, and Metastasis

The immune system is characteristically involved in the responses to tumor-specific antigens, a process like that is exerted against foreign pathogens during inflammation. However, one of the hallmarks of cancer is its ability to evade the immune system, and it is currently accepted that, when inflammation is prolonged over time, other mechanisms—still partially unknown—subsequently promote cancer progression [44]. Pancreatic cancer is a model of “cold” tumor and “immune privilege” (acquired or not). Despite the high mutational rate found in pancreatic cancer cells, only a moderate range of them are associated with the expression of neoepitopes, and this prevents the cancer cells from activating an effective adaptive immune response. The clinical relevance of the neoepitopes expression has been reported by Balachandran VP et al. [45], who compared rare long-term survivors (median survival of six years) with typical short-term survivors (median survival of 0.8 years), and demonstrated that the highest neoantigen number and the most abundant CD8^+^ T cell infiltrates are predictive of patients with the longest survival.

The mechanism underlying the ability of the immune system to initially protect a host from tumor growth and subsequent cancer progression, so called cancer immunoediting [46], resides mainly within the tumor and the surrounding stroma, which include fibroblasts, pancreatic stellate cells, and infiltrating immune cells. In this area, also known as the tumor microenvironment, the local immune response primes local inflammation, generated and maintained mainly by cytokines, chemokines, and other reactive molecules, such as reactive oxygen species (ROS) and small peptides. Local inflammation with NF-κB activation might also be induced by anti-cancer therapy, in particular Gemcitabine, which could act directly on the tumor microenvironment, or indirectly, by altering the equilibrium between the gut microbiota and the local inflammatory cells. In an animal model of PDAC, Gemcitabine was shown to shift the composition of the gut microbiome towards a pro-inflammatory phenotype. In the same model, Gemcitabine altered the serum metabolic profiling with decreased inosine, an immunosuppressive and anti-inflammatory adenosine metabolite, which overall favored a worsening of inflammation [47,48]. While cytokines, chemokines, and ROS derive mainly from infiltrating inflammatory cells, bioactive small peptides may result from the degradation of proteins by tumor-derived proteases that also cause extracellular matrix degradation [49]. The proximity of the cellular elements facilitates cross-talk between these mediators in the local inflammatory tumor-stroma microenvironment.

Cancer immunoediting mechanisms deserve greater attention, above all because the interactions originating in the tumor stroma are considered a major driver of the development of the ability of neoplastic cells to hijack the host inflammatory response, creating an environment that fosters tumor growth and progression [44]. Despite several inflammatory immune cell types being found in the PDAC dense stromal tissue, the microenvironment remains immunosuppressive in nature. This can be explained by the fact that the PDAC microenvironment restricts the infiltration of anti-tumor T-cells, whose role may be further attenuated by the co-infiltrating immune suppressive regulatory T-lymphocytes (T_reg_), myeloid derived suppressor cells (MDSC), and M2 macrophages. For example, MDSC induce T_reg_ to suppress T-lymphocyte activation in a cytokine-independent, but cell contact-dependent, manner. However, this phenomenon may also be due to the dense PDAC desmoplastic tissue, consisting mainly of fibroblasts and an extracellular matrix. The importance of these immune-suppressive cell types in PDAC is further supported by the clinical observation that they are associated with a poor prognosis [50]. Additionally, the mast cells are recruited by tumor stimuli and, on reaching the tumor microenvironment, can exert angiogenetic effects and release cytokines, thereby downregulating the immune response and favoring the expansion and activation of regulatory T cells, leading to immune tolerance [44].

Metastases, already present at diagnosis in more than half of PDAC patients, are the major cause of death following complete tumor resection, with metastatic recurrence being detected in about 70% of cases [51,52]. This highly metastatic burden and the associated poor survival depend on PDAC genetics, but also on the types of stromal infiltrating immune cells [53]. The relationship between PDAC genetics and inflammatory cells might underlie metastases through inflammatory mediators, for example by the calcium binding proteins S100A8 and S100A9. When the tumor suppressor gene *SMAD4* is expressed by PDAC cells, S100A8 and S100A9 are produced by the tumor infiltrating inflammatory cells. On the contrary, in the presence of homozygous *SMAD4* deletion, which occurs in about 50% of PDAC tumors, these inflammatory molecules are produced by PDAC cells themselves, not by infiltrating inflammatory cells [54,55]. S100A8 and S100A9 might act on distant sites, such as the liver or lung, where they can modify the microenvironment, thus creating a “pre-metastatic niche” favoring metastatic cells adhesion and growth [56,57].

## 4. Cytokines Released by Immune and Tumor Cells, and Their Role in Inflammatory Response to Cancer Cells

The role of cytokines, low-molecular weight proteins regulating many biological processes, including immunity, inflammation, metabolism, cell growth, and differentiation [58], is played both by modulating the tumor microenvironment and by directly affecting cancer cells. Several cytokines have a direct effect on cancer immunoediting and cancer progression. 

Tumor microenvironment inflammatory cells (e.g., immune cells, fibroblasts, and endothelial cells), but also cancer cells, are known to produce and secrete several cytokines, such as IL-6, IL-10, IL-13, VEGF, and TGFβ [59]. Within the tumor microenvironment, the balance between pro- and anti-inflammatory cytokines is continuously evolving, depending on the cross-talk between cancer cells and the inflammatory network cells. Among the vast bulk of the studied cytokines, a major role in PDAC is played by the anti-inflammatory TGFβ and IL-10, and by the pro-inflammatory IL-1β, IL-6, IL-17, and TNFα, of which the individual roles are summarized in Table 1. 

Cancer associated fibroblasts (CAFs) are an integral part of the tumor microenvironment. CAFs develop from bone marrow derived mesenchymal stem cells, quiescent resident fibroblasts, and pancreatic stellate cells. They attain an activated phenotype via various cytokines, while also function as a source of pro-inflammatory cytokines. The pathways that activate CAFs are Sonic Hedgehog, TGFβ, TNFα, IL-1, IL-6, and IL-10. CAFs produce most of the extracellular matrix, highly abundant in PDAC, but they also favor tumor growth and metastases by secreting inflammatory cytokines, mainly IL-6; chemokines; and chemokines ligands, namely CCL2 and C–X–C motif chemokine ligand 12 (CXCL12) [63,73,74,75].

Several studies have investigated the expression profile of various cytokines released in the sera (or other biological fluid) of patients with PDAC. In their recent systematic review, Yako et al. [59] found that at least 65 full text articles available in the literature report data on cytokines measured in patients’ serum/plasma, tissue, peripheral blood mononuclear cells (PBMC), pancreatic fluid, and whole blood. The authors found that most studies reported higher levels of IL-2, IL-8, IL-6, IL-10, macrophage inhibitory cytokine-1 (MIC-1), macrophage colony-stimulating factor (M-CSF), and VEGF in PDAC patients, than in reference individuals or pancreatitis patients. Conversely, there was little agreement between studies comparing TGFβ levels in PDAC patients and controls [59]. In one study, the serum levels of fibroblast growth factor 10/keratinocyte growth factor-2 (FGF-10/KGF-2), interferon inducible T cell alpha chemokine/chemokine C-X-C motif ligand 11 (I-TAC/CXCL11), oncostatin M (OSM), osteoactivin (OA)/glycoprotein nonmetastatic melanoma protein B (OA/GPNMB), and stem cell factor (SCF) were described as significantly increased in PDAC patients with respect to healthy controls [76]. In another study, the Panc02 murine PDAC cells line was shown to release, in culture media, high amounts of TGFβ and VEGF, of which the levels correlated with tumor progression when Panc02 cells were orthotopically injected in C57BL/6 mice, while levels of other cytokines, such as IL-1β and IL-2, did not vary [60].

A typical proinflammatory cytokine, IL-6, also has a pro-tumorigenic effect [70,62]. In vitro studies have shown that both pancreatic cancer cell lines and CAFs produce IL-6, which might act as an autocrine and paracrine stimulus causing increased tumor cell migration and invasion, as well as epithelial to mesenchymal transition (EMT) [60,63]. In PDAC patients, the elevated IL-6 levels found in serum, pancreatic juice and tissue, appear to predict tumor stage and survival, thus supporting the potential role of this cytokine as a malignancy predictor, but its sensitivity and specificity are extremely variable [59]. 

The role of TGFβ, a pleiotropic cytokine that has anti-inflammatory and immune-suppressive effects, is complex and paradoxical. Under physiological conditions, TGFβ has a well-documented role in cell proliferation and differentiation, while its effects may be different in cancer, depending on the stage of tumorigenesis. In the early stages, TGFβ may act as a tumor suppressing molecule, mainly by inhibiting cell cycle progression, but in later stages, it enhances invasion and metastasis by inducing epithelial to mesenchymal transition (EMT) [49].

IL-10, known to be a potent anti-inflammatory cytokine, can be secreted by almost all immune cells, and by tumor cells. On the one hand, IL-10 can inhibit NF-κB signaling, exerting an anti-tumour effect, but, on the other, because of its immunosuppressive effect, it can allow cell maturation and differentiation, thus fostering cancer immune-evasion [70].

TNFα, a master regulatory protein that plays a key role in the host immune response, is normally involved in systemic inflammation and fever [77]. TNFα is a type II transmembrane protein with signaling potential as a soluble cytokine, once released by proteolytic cleavage. While TNFα was initially described as a pro-inflammatory cytokine, a body of evidence currently supports the concept that it plays a dual role in carcinogenesis [70]. TNFα might play different biological roles, depending on the alternative engagement of its putative receptors, TNFR1 and TNFR2. TNFR1 (also known as p55, and is ubiquitously expressed) and TNFR2 (also known as p75, and is mainly expressed in immune cells) receptors are activated by the soluble or transmembrane form of TNFα, respectively. Once activated, they, in turn, trigger either apoptosis or NF-κB pathways. The different activation pathways have been evidenced by using knockout mice, in which a lack of TNFR1 causes a reduced inflammatory response and protects the animals from many diseases, while a lack of TNFR2 exacerbates conditions, such as poor survival following coronary artery ligation [78]. On the basis of its participation in chronic inflammatory disease, TNFα is believed to be implicated in several carcinogenetic processes, including colorectal cancer in patients with inflammatory bowel diseases and cholangiocarcinoma in patients with primary sclerosing cholangitis [70]. It has been suggested that this cytokine, especially when present in low concentrations, underlies tumor promotion, in the light of reactive oxygen species (ROS) and reactive nitrogen species (RNS), which can induce DNA damage. Accordingly, the pro- or anti-tumoral effect depends also on the local concentration and its expression site [70,79]. In the PDAC setting, it has been shown that not only immune cells, but also human pancreatic tumor cells, can produce and secrete TNFα at picogram levels. This suggests that PDAC cells are regularly exposed to their endogenous autocrine stimuli that determine, through the activation of NF-κB and Sonic Hedgehog pathways and the recruitment of T_reg_ at the cancer site, increased tumor cell invasiveness both in vitro and in vivo animal models [80,81,82]. The increased invasiveness of pancreatic cancer cells following TNFα treatment is also correlated with stemness and increased expression of the epidermal growth factor (EGF) receptor, these TNFα effects being potentiated by TGFβ [83]. The TNFα expression levels in human pancreatic cancer tissue was not correlated with tumor stage, but it was found to be associated with chemoresistance and to be of prognostic significance, a shorter survival being correlated with higher TNFα expression levels [72]. On the basis of the observed tumor promoting effects of TNFα, it was initially suggested that anti-TNFα antibodies might represent an effective anti-cancer therapy, and the ability of anti-TNFα antibodies to inhibit metastasis was demonstrated in late 1993 [79]. The reported effects of anti-TNFα treatment are heterogeneous. For example, in vitro anti-TNFα treatments have been shown to reduce PDAC cell viability, and to decrease the number of cellular elements and the amount of collagen in PDAC stroma. These findings support the hypothesis that the desmoplastic PDAC tumor stroma would be impaired by anti-TNFα treatments [72]. In vivo, mice repeatedly treated with infliximab or etanercept (biological treatment anti-TNFα) showed different effects, including a reduction in tumor growth and in liver metastases. However, of the biological anti-TNFα drug formulations, infliximab showed a stronger anti-tumor property than etanercept, especially in vivo [80]. Two clinical trials conducted in the United States evaluated the effects of TNFα and of TNFα inhibitors in patients with advanced pancreatic cancer. One, by Herman et al. [84], investigated the effect of TNFα delivered to tumor cells by gene transfer. This open-label, randomized, controlled phase III trial on unresectable pancreatic cancer patients included 187 patients treated with standard of care and TNFrade, and 90 standard of care only treated patients. The data on the overall survival, progression free survival, and time to progression demonstrated no difference between outcomes, following standard care, and standard care and TNFα. The other study, by Wu et al [85], evaluated whether combined anti-TNFα etanercept and gemcitabine improved the survival of patients with advanced pancreatic cancer with respect to gemcitabine alone. Like TNFα, anti-TNFα did not improve the survival. Therefore, although safe, TNFα and anti-TNFα therapy, provide no clinical benefit in terms of the duration of survival of patients with unresectable pancreatic cancer. However, the biological premises based on the above observation, that TNFα might be carcinogenic depending on dosage, reported in vitro and animal studies, poses the question as to whether patients treated with anti-TNFα are at risk of pancreatic cancer. The Food and Drug Administration (FDA) and European Medicines Agency (EMA) have approved five anti-TNFα biologics (infliximab, adalimumab, golimumab, etanercept, nd certolizumab), which inhibit or modulate the effects of TNFα, in the treatment of rheumatic (rheumatoid arthritis, anckylosing spondylitis, and psoriasic arthritis) and inflammatory bowel diseases. Studies in the literature conclude that anti-TNFα treatment is not associated with increased cancer risk in the short-term, although there is consensus on the fact that long-term risk assessment calls for observational studies [86,87,88]. Interestingly, although rarely reported, new onset PDAC in patients treated with anti-TNF has been described [89].

Cytokines and drugs targeting their pathways might enhance or reduce cancer risk and cancer progression by directly acting on tumor cells and/or on immune cells. However, other potential mechanisms might be also involved, these including those evoked by microRNA (miRNA), short non-coding RNA sequences that regulate mRNA translation. In PDAC, up-regulated oncogenic miRNA and down-regulated tumor suppressor miRNA have been associated with tumor growth and metastases [90]. A comprehensive reference source for miRNA altered in human PDAC is given by the DBDEMC2 database, which reports the results on differently expressed miRNA in human cancers detected by high throughput methods [91]. miRNA expression levels might be enhanced or dampened by cytokines and growth factors, and, on the other hand, miRNA might regulate the expression of cytokines, growth factors, and of their receptors. In experiments made by using PDAC cell lines, Ottaviani et al. recently demonstrated that TGFβ induces the expression of oncogenic miR-100 and miR-125b, but not that of the anti-tumorigenic let-7a family [92], while we have previously demonstrated that the epidermal growth factor (EGF) induces the expression of miR-133a and miR-99 [93]. Based on the hypothesis that anti-TNFα therapy might de-regulate miRNA expression, we first analyzed those studies that evaluated the miRNA expression in patients treated with anti-TNFα, and then compared the de-regulated miRNA with the PDAC findings reported in the DBDEMC2 database (see Table 2). Some studies in the literature report on the up- or down-regulation of miRNA following anti-TNFα therapy [94,95,96,97,98,99,100]. Notably, some miRNA (i.e., hsa-let-7d, hsa-miR-221, hsa-miR-224, hsa-miR-23a, and hsa-miR-197) were found to be up-regulated by anti-TNFα, and are reportedly associated with pancreatic cancer [91,101]. This finding further supports the need for caution in evaluating anti-TNFα therapy.

## 5. Cytokines Are Involved in Cachexia and Cancer Induced Metabolic Alterations 

Several pro-inflammatory cytokines have also been reported to be associated with the clinical effects of inflammation; fever, an important paraneoplastic effect, is believed to be due to increased concentrations of circulating IL-6, TNFα, and IL-1β [102]. Cancer cachexia syndrome, the most extreme known consequence of systemic inflammation, frequently affects patients with advanced cancer. This syndrome, characterized by muscle tissue wasting, causes atrophy and mobility impairment because of fatigue and weakness [103]. While TNFα is known to play a major role in cancer-associated cachexia in general, the loss of exocrine pancreas function is one of the main causes of malnutrition and weight loss in cases of PDAC. Pre-clinical studies suggest that TNFα plays a major role in cancer cachexia, above all in muscle wasting. In mice, the peritoneal injection of cancer cells expressing TNFα has been shown to cause weight loss and cachexia [103]. The effect of TNFα on protein kinase C, for example, leads to the rapid conjugation of ubiquitin to muscle proteins, eventually leading to the enhancement of the proteosomal degradation of cellular proteins [104]. 

However, cachexia is accompanied by metabolic alterations triggered by deregulated carbohydrate and lipid metabolism, which, in turn, increase energy expenditure. For example, the marked metabolic alteration found in the tumor and non-tumor tissue of many cancer patients results mainly in glucose overconsumption and excessive lactate production. It has been demonstrated that TNFα can activate two key regulatory glycolysis enzymes, namely phosphofructokinase and fructose-1.6-bisposphatase. Moreover, small peptides derived from, for example, the proteolytic cleavage of full molecules, have been shown to alter not only PDAC metastatic potential by increasing cell growth and invasion, but also to modify β-cells insulin release by impairing the calcium flow [105].

**Figure 1:**
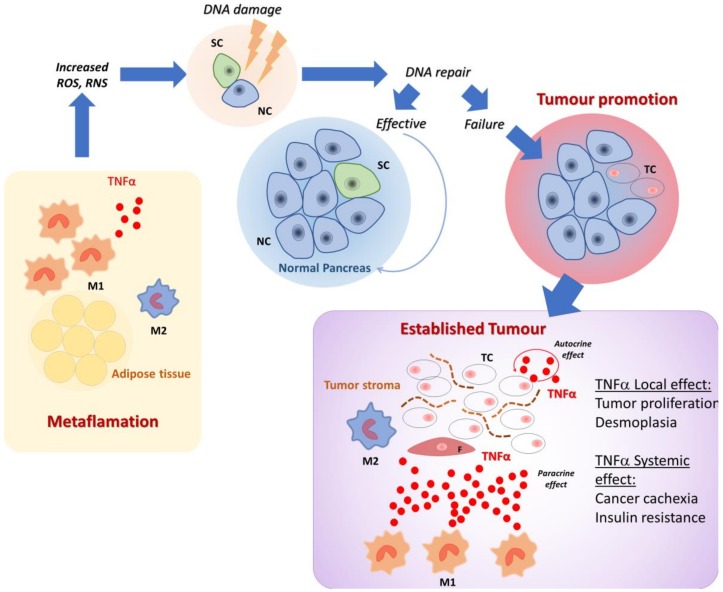
Tumor necrosis factor alpha (TNFα) role in tumor promotion and in tumor-associated inflammation. In adiposity-associated systemic inflammation (metaflammation), pro-inflammatory M1 macrophages infiltrate the adipose tissue prevailing over M2 macrophages. Low amounts of TNFα, produced by M1 macrophages, might cause DNA damage by inducing reactive oxygen (ROS) and nitrogen (RNS) species. When normal pancreatic ductal cells (NC), but mainly stem cells (SC) are targeted, the failure of DNA repair after damage results in the clonal expansion of tumor cells (TC) and tumor establishment. Within the tumor microenvironment, made of stroma, tumor-associated fibroblasts (F), and inflammatory cells, including M1 and M2 macrophages, TNFα is produced by both TC and M1 macrophages. TNFα exerts effects on TC, inducing proliferation; on F, inducing stroma production and desmoplasia; and on distant sites concurring in causing cancer-associated cachexia and insulin resistance.

Insulin resistance has also been suggested as a relevant mechanism involved in cancer cachexia. TNFα has been cited as being responsible for decreasing insulin sensitivity and contributing to insulin resistance in cancer [104]. Figure 1 shows the roles of TNFα in tumor promotion and in local tumor-associated inflammation.

## 6. Immune Response to Cancer Cells

Tumor-associated inflammation, a dynamic process, involves the infiltration of multiple immune cells subtypes into the tumor stroma. It is now known that both the innate and the adaptive immune systems are active against human cancer. Consequently, several studies have focused on the role of the immune system in the onset and progression of cancer [46,106]. The innate immune system consists of immune cells, already present in the body, that can be immediately recruited to the site of infection [46]. Innate immune cells include granulocytes, macrophages, mast cells, and natural killer (NK) and dendritic (DC) cells. Neutrophils usually trigger a prompt response to inflammation and can secrete cytokines, whereas after maturation, macrophages can differentiate into either M1, which can trigger the inflammatory response, and M2-polarized cells, which usually restrain the inflammatory response and promote tumor growth [46]. MDSC represent another group of innate immune cells that suppress both innate and adaptive immunity. 

### 6.1. Myeloid-Derived Suppressor Cells

MDSC comprise a heterogeneous population of immature myeloid cells that derive from common precursors of DC, macrophages, and granulocytes [60]. In healthy organisms, the natural immune function of MDSC is to suppress inflammation by inhibiting both T and NK lymphocytes. However, in cancer patients, MDSC are overproduced by bone marrow, being mobilized into tumor stroma, their abnormal accumulation potentially promoting immunosuppression and immune evasion, thus favoring cancer immunoediting [32,46,60,107]. It has been demonstrated that MDSC levels are higher in PDAC patients than in healthy subjects, and this increase correlates with augmented CD4^+^ Th2 and T_reg_ lymphocyte levels [106,108]. 

The different growth factors suggested to be implicated in MDSC expansion include IL-13 and granulocyte macrophage colony stimulating factor (GM-CSF), although the latter has been more widely studied and well documented [108]. Despite the fact that GM-CSF is normally produced by the organism in response to infection, in order to boost the innate immune response, increased GM-CSF levels cause myeloid progenitor cells in bone marrow to differentiate into MDSC, to migrate into the circulation, and accumulate in both the tumor and the spleen [107,108,109]. Hypoxia has been proposed as a key mediator in the recruitment of MDSC in the tumor microenvironment. Indeed, PDAC, well known for its thick, poorly vascularized extracellular matrix, causes a hypoxic environment that is more advantageous for the tumor than for host cells. However, this deprivation causes the expression and upregulation of hypoxia-induced factors (HIF) that contribute to the recruitment of MDSC [108]. Recently, MDSC have been divided into monocytic and granulocytic subsets, reflecting differential expressions of Ly6C and Ly6G markers [110]. 

Once within the tumor microenvironment, MDSC may have an antigen-nonspecific and antigen-specific immunosuppressive effect [108]. The antigen-nonspecific effect is mediated by the release of oxygen species within the tumor microenvironment, which causes oxidative stress in the surrounding immune cells and inhibits T cell proliferation. The antigen-specific effect alters the ability of MDSC to take up and process antigens, inducing immune tolerance in CD8^+^ T effector cells [108]. 

Although the main triggers for MDSCs recruitment and differentiation are inflammatory cytokines and growth factors, it is now emerging that other factors, besides oxygen and including stromal pH and nutrients, might shape these cells. The role of nutrients appears of particular relevance and further supports the notion that glucose and lipid metabolism could impact not only PDAC risk, but also PDAC growth and progression. The accumulation in the tumor microenvironment of lactate, the main metabolite of tumor cell aerobic glycolysis, induces MDSC expansion, thus favoring immunosuppression, with MDSC exhibiting an increased uptake of fatty acids associated with the increased expression of fatty acid oxidation enzymes [32,111,112].

### 6.2. Tumour Associated Macrophages (TAM)

Alongside MDSC, macrophages, another type of immune cell, play an important role in acute and chronic inflammation. Normally responsible for clearing debris from sites of injury or infection, they can also present antigens to the hosts’ immune cells (B and T cells), triggering adaptive immunity. Furthermore, they can undergo a differentiation into two phenotypes, M1 and M2, similar to the classification scheme of CD4^+^T cells into Th1 and Th2. One of the most important cytokines expressed by the M1 pro-inflammatory profile is TNFα, whereas the anti-inflammatory phenotype M2 produces the immunosuppressive cytokine IL-10 [108]. Although the amount of each phenotype can change during tumor development, all tumors contain M1 and M2 TAM subtypes. It has been shown that, in early stage cancer, TAM exhibits more of a proinflammatory M1 phenotype that promotes antitumor activity, whereas as the disease progresses, they exhibit more of an M2 phenotype, which is anti-inflammatory and contributes to tumor immunoediting, fostering tumor growth and invasion. Experimental studies performed in bone-marrow-derived macrophages, obtained after stimuli with recombinant macrophage colony stimulating factor 1 (CSF-1), IL-4, and IL-13, showed that TNFα blocks a set of M2 gene expression [71]. TNFα also suppresses IL-13 expression by eosinophils, thus preventing the production of a key M2-activating cytokine. Further evidence of the central role played by TNFα in the M2 phenotype polarization was obtained by means of etanercept, a soluble TNFR that partially limits TNFα bioavailability. The administration of this anti-TNFα therapy to mice bearing ovary EG7 tumors was followed by the increased IL-13 and M2 gene expression of TAM [71]. It was recently demonstrated that the close association between the master inflammatory NF-κB signaling and TNFα regulates the delicate balance between TAM and pancreatic cancer cells during the early stages of carcinogenesis [113]. In pancreatic cells, the activation of NF-κB promotes the production of a TGFβ superfamily member, GDF-15, which acts on TAM by inhibiting NF-κB signaling. GDF-15 reduces TNFα synthesis, and is followed by reduced TNFα-dependent tumor cell apoptosis and enhanced tumor growth [113]. 

### 6.3. T_reg_ Cells

The presence of T_reg_ cells, a prominent component of the immune infiltrate on the tumor stroma, correlates with a poor clinical outcome in many cancer types [114]. T_reg_ cells, considered the most potent known inhibitors of antitumor immunity, can downgrade the activity of CD4^+^, CD8^+^, and NK cells. The various mechanisms suggested as underlying T_reg_ cell-mediated immune suppression include the direct elimination of, or competition with, effector T cells for access to antigen-presenting cells. The release of TGFβ and IL-10 from T_reg_ has also been postulated as a possible mechanism causing the immune suppression role of this cell type. Nevertheless, the exact role of T_reg_ cells in pancreatic tumorigenesis remains largely unknown. Jan et al. demonstrated that T_reg_ can confer an immunosuppressive property to their critical target, CD11c^+^ DC, which suppress immunity against cancer cells [114]. Additionally, the adhesion molecule L1CAM (CD171) is upregulated in the pancreatic ductal epithelium during PDAC progression, in association with the accumulation of immunosuppressive T cells in tumor stroma [115].

From a clinical viewpoint, the overall number of circulating white blood cells and platelets might have prognostic implications in PDAC patients. A poor prognosis in these subjects is usually associated with a reduced lymphocyte count and increased platelet and polymorphonuclear cell counts. The derived lymphocyte/monocyte, neutrophil/lymphocyte, and platelet/lymphocyte ratios have been proposed as prognostic indices, their utility being supported by findings made in several studies [116,117,118].

## 7. Conclusions

Inflammation and metaflammation increase the risk of pancreatic cancer, but pancreatic cancer induces an immunosuppressive inflammatory reaction. The equilibrium between inflammatory cells-derived and cancer cell-derived cytokines and chemokines influences both carcinogenesis and the cancer associated inflammatory reaction. In this context, TNFα, produced by the tumor cells themselves and by tumor-infiltrating inflammatory cells, is involved in carcinogenesis, tumor progression, metastases, and anti-cancer immune control. Although inflammation remains a suitable target for therapy, the focus should be on emerging therapies targeting inflammatory cytokines, not only for their potential anti-tumor, but also for their potential pro-tumor effects. 

## Figures and Tables

**Table 1:** Cytokines in pancreatic cancer.

***Pro-Inflammatory Cytokines***				
**Cytokine**	**Cell sources**	**PDAC cells production in vitro**	**Effects on immune cells**	**Effects on PDAC initiation and EMT**
TGF [58,60,61,62,63]	M2 macrophages, Th2 lymphocytes, and TAM	Yes—high	Promotes immune evasion and tolerogenic DC	Inhibits cell cycle progression in early stages, enhances invasion and metastasis by inducing EMT in advanced stages
IL-10 [60,61]	M2 macrophages, T_reg_, Mast cells, and TAM	Yes—high	Promotes immune evasion	PDAC associated TAM have a mixed M1 and M2 phenotype, produce high amounts of IL-10, IL-1β, IL-6 and TNFα and induce EMT in early tumorigenesis
***Pro-Inflammatory Cytokines***				
**Cytokine**	**Cell sources**	**PDAC cells production in vitro**	**Effects on immune cells**	**Effects on PDAC initiation and EMT**
IL-6 [58,60,62,63]	CAFs and TAM	Yes—high	Promotes Th2 type cytokine production	Promotes oncogenesis through JAK2-STAT3 activation, angiogenesis through the induction of VEGF, cancer cell migration and EMT
IL-1β [58,60,61,64,65]	DC, M1 macrophages, and TAM	Yes—low	Recruitment of MDSC and T-cell activation by inducing the production of IL-2 and IL-2R	Promotes cancer growth, invasion, and metastases
IL-17 [60,66,67,68,69]	Th 17 CD4^+^ cells	Yes—low	Recruitment of MDSC	Induces stemness, tumor initiation, and progression, not complete EMT. The expression of the IL-17 receptor is evident on cancer cells undergoing EMT, and depends on oncogenic Kras
TNFα [46,60,61,70,71,72]	M1 macrophages, TAM, neutrophils, mast cells, and pancreatic stellate cells	Yes—low	Antagonizes M2 macrophages polarization	Associated with PDAC initiation. Promotes angiogenesis by inducing VEGF production by fibroblasts and metastases by activating NF-β signaling

CAFs—cancer associated fibroblasts; DC—dendritic cells; EMT—epithelial to mesenchymal transition; MDSC—myeloid derived suppressor cells; PSCs—pancreatic stellate cells; TAM—tumor-associated macrophages. VEGF—vascular endothelial growth factor; TGFβ—transforming growth factor beta; IL—interleukin; TNF—tumor necrosis factor; PDAC—pancreatic ductal adenocarcinoma.

**Table 2:** Anti-TNFα, miRNA de-regulated expression, and PDAC. To construct the table, studies that evaluated the effects of anti-TNFα therapy on miRNA expression were first selected. Secondly, those on miRNA that reported to be also associated with PDAC were chosen and detailed in the table. Only the RT-PCR-validated or statistically significant miRNA were used for the comparison.

miRNA	Up or Down-Regulated in PDAC [91] *	Average logFold Change *	Up or Down-Regulated by Anti TNFα Therapy	Anti-TNFα Therapy
**hsa-miR-146 # polymorphism**	Found downregulated in one study *	-	No correlation	Crohn’s disease treated with infliximab or adalimumab [94].
**hsa-miR-196a # polymorphism**	Found upregulated in three studies *		No correlation	
**hsa-miR-221 polymorphism**	Found upregulated in five studies	1.9	No correlation	
**hsa-miR-224 polymorphism**	Found upregulated in four studies	1.8	No correlation	
**hsa-miR-106b**	Found upregulated in seven studies cancer/normal	1.33	Downregulated in treated patients	Psoriasis treated with etanercept [95].
**hsa-miR-26b**	Found upregulated in one study/downregulated in three studies	−0.9	Downregulated in treated patients	
**hsa-miR-143-3p**	-		Downregulated in treated patients	
**hsa-miR-223**	Found upregulated in three studies/downregulated in one study	1.11	Downregulated in treated patients	
**hsa-miR-126**	Found upregulated in one study/downregulated in one study	−2.41	Downregulated in treated patients	
**hsa-miR-5196**	Not found	-	Downregulated in treated patients	Rheumatoid arthritis and ankylosing spondylitis treated with anti-TNFα treatment (Golimumab in 15%, Adalimumab in 77%, Certolizumab in 8%) [96].
**hsa-miR-125b #**	Found upregulated in two studies/downregulated in three studies *	−0.28	Upregulated in treated patients	Rheumatoid arthritis treated with infliximab or etanercept or adalimumab [97].
**hsa-miR-126-3p**	Found downregulated in one study	−0.70	Upregulated in treated patients	
**hsa-miR-146a-5p**	Found downregulated in one study	−0.22	Upregulated in treated patients (see above)	
**hsa-miR-16-5p**	Found downregulated in one study	−0.92	Upregulated in treated patients	
**hsa-miR-23-3p**	Not found	-		
**hsa-miR-223-3p**	Found downregulated in one study	−1.17	Upregulated in treated patients	
**hsa-miR-22**	Found upregulated in one study	1.23	Downregulated in Adalimumab respondent patients	Rheumatoid arthritis treated with Adalimumab [98].
**hsa-miR-886-3p**	-	-	Upregulated in Adalimumab respondent patients	
**hsa-let-7d**	Found upregulated in 3 studies/Downregulated in 1 study	0.54	Upregulated in treated patients	Crohn’s disease treated with infliximab [99].
**hsa-let-7e**	Found upregulated in 1 studies/Downregulated in 1 study	0.33	Upregulated in treated patients	
**hsa-miR-28-5p**	Found upregulated in 2 studies/Downregulated in 1 study	0.64	Upregulated in treated patients	
**hsa-miR-221**	Found upregulated in 5 studies	1.9	Upregulated in treated patients (see above)	
**hsa-miR-224**	Found upregulated in 4 studies	1.8	Upregulated in treated patients (see above)	
**hsa-miR-99a**	Found upregulated in 2studies/Downregulated in 3 studies	−0.60	Upregulated in Adalimumab respondent	Rheumatoid arthritis treated with adalimumab or etanercept [100].
**hsa-miR-143**	Found upregulated in 3 studies/Downregulated in 2 studies	−1.05	Downregulated in respondent	
**hsa-miR-23a**	Found upregulated in 6 studies	1.29	Upregulated in respondent	
**hsa-miR-197**	Found upregulated in 5 studies/Downregulated in 1 study	1.20	Upregulated in respondent	

* In the DBDEMC2 database, cancers were compared with healthy controls. ^#^ In miRbase (http://www.mirbase.org), hsa-miR-146 corresponded to hsa-miR-146-5p, hsa-miR-196a corresponded to hsa-miR-196a-5p, and hsa-miR-125b corresponded to hsa-miR-125b-5p [101].
